# Supplementary material for: Deletion of wfs1 Impairs Oligodendrocyte Precursor Cells Dorsal Distribution and Myelination Through the wfs1-hmgcs1 Axis in Zebrafish
Source: Biology (Basel). 2026 Aug 16;15(16):1402. doi: 10.3390/biology15161402 (PMC13510151; doi:10.3390/biology15161402)
Supplement: Supplementary file 1 [file biology-15-01402-s001.zip › File S1-Transcriptome sequencing data and differentially expressed genes (DEGs) in wfs1-- zebrafish at 5 dpf/2.KEGG/wfs1KOvsWT/GSEA/wfs1KO/CELL_CYCLE/CELL_CYCLE(DRE04110).html]

Details for gene set CELL\_CYCLE(DRE04110)[GSEA]

|  || Dataset | deg.sample |
| Phenotype | sample.cls |
| Upregulated in class | wfs1KO |
| GeneSet | CELL\_CYCLE(DRE04110) |
| Enrichment Score (ES) | 0.23333094 |
| Normalized Enrichment Score (NES) | 1.3502839 |
| Nominal p-value | 0.0 |
| FDR q-value | 0.25921854 |
| FWER p-Value | 0.931 |
Table: GSEA Results Summary

  

Fig 1: Enrichment plot: CELL\_CYCLE(DRE04110)      
 Profile of the Running ES Score & Positions of GeneSet Members on the Rank Ordered List

  

| PROBE | DESCRIPTION (from dataset) | GENE SYMBOL | GENE\_TITLE | RANK IN GENE LIST | RANK METRIC SCORE | RUNNING ES | CORE ENRICHMENT || 1 | 327226 | casc5 |  |  | 71 | 4.814 | 0.0337 | Yes |
| 2 | 560751\_1 | stag2b |  |  | 176 | 4.349 | 0.0630 | Yes |
| 3 | 566841 | crebbpa |  |  | 530 | 2.904 | 0.0735 | Yes |
| 4 | 431763 | gadd45ab |  |  | 713 | 2.560 | 0.0869 | Yes |
| 5 | 327060 | wee1 |  |  | 928 | 2.192 | 0.0965 | Yes |
| 6 | 554509\_1 | orc1 |  |  | 942 | 2.178 | 0.1124 | Yes |
| 7 | 767751\_1 | ccnb3 |  |  | 975 | 2.119 | 0.1272 | Yes |
| 8 | 559723 | tgfb2l |  |  | 1105 | 1.990 | 0.1380 | Yes |
| 9 | 100141368\_1 | waplb |  |  | 1265 | 1.836 | 0.1467 | Yes |
| 10 | 280649\_1 | plk1 |  |  | 1335 | 1.777 | 0.1578 | Yes |
| 11 | 562282 | ppp2r5b |  |  | 1401 | 1.724 | 0.1687 | Yes |
| 12 | 100151416 | cdkn1a |  |  | 1627 | 1.584 | 0.1734 | Yes |
| 13 | 324475 | smc3 |  |  | 2204 | 1.283 | 0.1648 | Yes |
| 14 | 101886806 | mad2l1bp |  |  | 2279 | 1.256 | 0.1719 | Yes |
| 15 | 794108\_1 | nipblb |  |  | 2332 | 1.236 | 0.1795 | Yes |
| 16 | 768161 | orc2 |  |  | 2365 | 1.221 | 0.1876 | Yes |
| 17 | 393548 | gadd45aa |  |  | 2430 | 1.197 | 0.1945 | Yes |
| 18 | 323883\_1 | cul1a |  |  | 2816 | 1.080 | 0.1904 | Yes |
| 19 | 30590\_1 | tp53 |  |  | 2944 | 1.051 | 0.1943 | Yes |
| 20 | 565038 | ccnd2b |  |  | 3069 | 1.021 | 0.1980 | Yes |
| 21 | 497646 | gadd45bb |  |  | 3089 | 1.017 | 0.2050 | Yes |
| 22 | 327471\_1 | wee2 |  |  | 3241 | 0.984 | 0.2076 | Yes |
| 23 | 393554 | trip13 |  |  | 3321 | 0.968 | 0.2123 | Yes |
| 24 | 406582 | ppp2cb |  |  | 3436 | 0.944 | 0.2158 | Yes |
| 25 | 100034507 | cdk6 |  |  | 3458 | 0.939 | 0.2221 | Yes |
| 26 | 568795 | anapc1 |  |  | 3768 | 0.875 | 0.2189 | Yes |
| 27 | 550434 | mad2l1 |  |  | 3877 | 0.853 | 0.2218 | Yes |
| 28 | novel.211 | - |  |  | 3961 | 0.837 | 0.2255 | Yes |
| 29 | 564982\_2 | mcm6l |  |  | 4088 | 0.814 | 0.2276 | Yes |
| 30 | 797406\_3 | espl1 |  |  | 4099 | 0.811 | 0.2333 | Yes |
| 31 | 30637\_1 | mdm2 |  |  | 4722 | 0.715 | 0.2190 | No |
| 32 | 564533 | stag1a |  |  | 4778 | 0.708 | 0.2226 | No |
| 33 | 101885420 | e2f1 |  |  | 4903 | 0.693 | 0.2239 | No |
| 34 | 406353 | cdc20 |  |  | 5024 | 0.679 | 0.2251 | No |
| 35 | 492768 | cdc7 |  |  | 5323 | 0.645 | 0.2205 | No |
| 36 | 553345 | wapla |  |  | 5405 | 0.640 | 0.2228 | No |
| 37 | 791138 | sgol1 |  |  | 5708 | 0.616 | 0.2178 | No |
| 38 | 559398 | cdca5 |  |  | 6220 | 0.566 | 0.2059 | No |
| 39 | 562460 | ppp2r5a |  |  | 6255 | 0.562 | 0.2091 | No |
| 40 | 777733 | rb1 |  |  | 6262 | 0.561 | 0.2131 | No |
| 41 | 553345\_1 | wapla |  |  | 6300 | 0.557 | 0.2161 | No |
| 42 | 58092\_1 | smad3a |  |  | 6307 | 0.555 | 0.2200 | No |
| 43 | 402908 | e2f5 |  |  | 6362 | 0.551 | 0.2224 | No |
| 44 | 406304 | gadd45ba |  |  | 6674 | 0.522 | 0.2165 | No |
| 45 | 559273\_1 | ep300a |  |  | 6707 | 0.519 | 0.2194 | No |
| 46 | 492353 | ddx11 |  |  | 6808 | 0.510 | 0.2200 | No |
| 47 | 30654 | gsk3b |  |  | 6859 | 0.504 | 0.2222 | No |
| 48 | 30686 | myca |  |  | 6877 | 0.502 | 0.2254 | No |
| 49 | 57925 | cdc25b |  |  | 6892 | 0.501 | 0.2287 | No |
| 50 | 402991 | gadd45ga |  |  | 6915 | 0.499 | 0.2318 | No |
| 51 | 317763 | ttk |  |  | 7143 | 0.478 | 0.2282 | No |
| 52 | 100151181 | si:dkey-239n17.4 |  |  | 7340 | 0.463 | 0.2254 | No |
| 53 | 445395 | esco2 |  |  | 7438 | 0.458 | 0.2258 | No |
| 54 | 322275 | rad21a |  |  | 7470 | 0.455 | 0.2282 | No |
| 55 | 567168 | stag2a |  |  | 7772 | 0.439 | 0.2220 | No |
| 56 | 100333282 | esco1 |  |  | 7957 | 0.426 | 0.2194 | No |
| 57 | 286747 | mcm5 |  |  | 8517 | 0.401 | 0.2047 | No |
| 58 | 100000720 | abl1 |  |  | 8525 | 0.401 | 0.2075 | No |
| 59 | 337598 | mcm4 |  |  | 8592 | 0.396 | 0.2084 | No |
| 60 | 100049158 | pkmyt1 |  |  | 8609 | 0.394 | 0.2108 | No |
| 61 | 445392 | fbxo5 |  |  | 8652 | 0.391 | 0.2124 | No |
| 62 | 368316 | ccnb2 |  |  | 8799 | 0.381 | 0.2106 | No |
| 63 | 100307086\_1 | pttg1 |  |  | 9025 | 0.366 | 0.2063 | No |
| 64 | 795522 | rbl1 |  |  | 9490 | 0.335 | 0.1941 | No |
| 65 | 566409 | mcm6 |  |  | 9603 | 0.327 | 0.1930 | No |
| 66 | 192323 | mcm3 |  |  | 9643 | 0.324 | 0.1942 | No |
| 67 | 563884 | tgfb1b |  |  | 9725 | 0.320 | 0.1940 | No |
| 68 | 406507 | gadd45gb.1 |  |  | 9742 | 0.319 | 0.1959 | No |
| 69 | 791146 | e2f3 |  |  | 9746 | 0.319 | 0.1982 | No |
| 70 | 567006 | bub1bb |  |  | 10023 | 0.302 | 0.1917 | No |
| 71 | 560317 | si:dkey-222b8.1 |  |  | 10062 | 0.300 | 0.1928 | No |
| 72 | 30188\_1 | ccne1 |  |  | 10089 | 0.297 | 0.1942 | No |
| 73 | 393162 | chek1 |  |  | 10187 | 0.291 | 0.1933 | No |
| 74 | 323883\_2 | cul1a |  |  | 10317 | 0.284 | 0.1913 | No |
| 75 | 393141 | mycb |  |  | 10475 | 0.274 | 0.1884 | No |
| 76 | 406813\_1 | tfdp1a |  |  | 10570 | 0.268 | 0.1875 | No |
| 77 | 373881 | ppp2r5ea |  |  | 10578 | 0.268 | 0.1893 | No |
| 78 | 415165 | ccne2 |  |  | 10615 | 0.265 | 0.1901 | No |
| 79 | 369194 | tgfb2 |  |  | 10813 | 0.254 | 0.1858 | No |
| 80 | 324475\_2 | smc3 |  |  | 10821 | 0.254 | 0.1875 | No |
| 81 | 336998\_1 | chek2 |  |  | 11434 | 0.220 | 0.1698 | No |
| 82 | 791894 | aurkb |  |  | 11616 | 0.210 | 0.1656 | No |
| 83 | 100147915 | mcm3l |  |  | 11619 | 0.210 | 0.1671 | No |
| 84 | 326283 | smad3b |  |  | 11759 | 0.203 | 0.1643 | No |
| 85 | 322060\_1 | ywhae1 |  |  | 11839 | 0.199 | 0.1632 | No |
| 86 | 565592 | pds5b |  |  | 12030 | 0.189 | 0.1587 | No |
| 87 | 562283 | prkdc |  |  | 12074 | 0.187 | 0.1587 | No |
| 88 | 100005981 | ppp2r5ca |  |  | 12095 | 0.185 | 0.1594 | No |
| 89 | 393907 | cdc23 |  |  | 12381 | 0.170 | 0.1517 | No |
| 90 | 368329 | cdkn1bb |  |  | 12428 | 0.168 | 0.1515 | No |
| 91 | 100004175 | cdkn1cb |  |  | 12458 | 0.167 | 0.1518 | No |
| 92 | 794669 | gb:cr929477 |  |  | 12490 | 0.165 | 0.1521 | No |
| 93 | 323055 | ywhaba |  |  | 12517 | 0.163 | 0.1525 | No |
| 94 | 445501\_1 | cdc26 |  |  | 12719 | 0.153 | 0.1473 | No |
| 95 | 406582\_1 | ppp2cb |  |  | 12771 | 0.151 | 0.1468 | No |
| 96 | 494046 | anapc4 |  |  | 12917 | 0.145 | 0.1433 | No |
| 97 | 402862 | cdkn1ba |  |  | 12928 | 0.144 | 0.1441 | No |
| 98 | 393148 | cdc14b |  |  | 12989 | 0.141 | 0.1432 | No |
| 99 | 393223 | fzr1a |  |  | 13089 | 0.136 | 0.1411 | No |
| 100 | 30450 | cdc27 |  |  | 13112 | 0.135 | 0.1414 | No |
| 101 | 80973 | cdk1 |  |  | 13334 | 0.124 | 0.1354 | No |
| 102 | 406715 | cdk2 |  |  | 13336 | 0.124 | 0.1363 | No |
| 103 | 192295 | ccna2 |  |  | 13449 | 0.118 | 0.1336 | No |
| 104 | 567111 | crebbpb |  |  | 13470 | 0.117 | 0.1339 | No |
| 105 | 797354 | smc1b |  |  | 13508 | 0.115 | 0.1335 | No |
| 106 | 566753 | dbf4b |  |  | 13517 | 0.115 | 0.1341 | No |
| 107 | 557208 | ppp2r5cb |  |  | 13564 | 0.112 | 0.1335 | No |
| 108 | 566787 | cdt1 |  |  | 13621 | 0.110 | 0.1326 | No |
| 109 | 798352 | anapc10 |  |  | 13844 | 0.100 | 0.1263 | No |
| 110 | 403012 | bub3 |  |  | 14162 | 0.084 | 0.1169 | No |
| 111 | 550585 | ppp2ca |  |  | 14235 | 0.081 | 0.1153 | No |
| 112 | 192338 | mcm2 |  |  | 14301 | 0.078 | 0.1138 | No |
| 113 | 566831 | rad21b |  |  | 14452 | 0.072 | 0.1096 | No |
| 114 | 393716 | skp1 |  |  | 14456 | 0.071 | 0.1100 | No |
| 115 | 399487 | ywhaqa |  |  | 14509 | 0.069 | 0.1089 | No |
| 116 | 321729 | ywhabl |  |  | 14700 | 0.060 | 0.1034 | No |
| 117 | 58025 | ccnb1 |  |  | 14783 | 0.056 | 0.1012 | No |
| 118 | 100148604 | fzr1b |  |  | 14885 | 0.052 | 0.0984 | No |
| 119 | 100534898 | mtbp |  |  | 14964 | 0.048 | 0.0963 | No |
| 120 | 563669 | stag1b |  |  | 15122 | 0.040 | 0.0916 | No |
| 121 | 334315 | orc3 |  |  | 15181 | 0.038 | 0.0901 | No |
| 122 | 550576 | anapc16 |  |  | 15266 | 0.034 | 0.0877 | No |
| 123 | 555797 | cdkn2c |  |  | 15302 | 0.032 | 0.0868 | No |
| 124 | 768140\_1 | anapc11 |  |  | 15393 | 0.028 | 0.0842 | No |
| 125 | 393886 | mad1l1 |  |  | 15558 | 0.019 | 0.0791 | No |
| 126 | 565612 | ep300b |  |  | 15569 | 0.019 | 0.0790 | No |
| 127 | 799608 | ccnd2a |  |  | 15743 | 0.009 | 0.0736 | No |
| 128 | 393967 | pds5a |  |  | 15806 | 0.006 | 0.0717 | No |
| 129 | 562014\_1 | cdc16 |  |  | 15870 | 0.003 | 0.0697 | No |
| 130 | 322060 | ywhae1 |  |  | 15887 | 0.003 | 0.0692 | No |
| 131 | 445411 | ticrr |  |  | 16257 | -0.009 | 0.0576 | No |
| 132 | 406695 | cdc45 |  |  | 16335 | -0.012 | 0.0553 | No |
| 133 | 559111 | smad4a |  |  | 16436 | -0.016 | 0.0522 | No |
| 134 | 565969 | cdc14ab |  |  | 16437 | -0.016 | 0.0524 | No |
| 135 | 559273 | ep300a |  |  | 16774 | -0.025 | 0.0419 | No |
| 136 | 768140 | anapc11 |  |  | 16783 | -0.026 | 0.0419 | No |
| 137 | 550258 | si:dkey-23c22.2 |  |  | 16859 | -0.029 | 0.0397 | No |
| 138 | 406685 | ppp2r1ba |  |  | 16873 | -0.029 | 0.0395 | No |
| 139 | 564967 | zbtb17 |  |  | 17178 | -0.040 | 0.0302 | No |
| 140 | 403060 | smc1al |  |  | 17273 | -0.044 | 0.0276 | No |
| 141 | 570900 | nipbla |  |  | 17590 | -0.058 | 0.0180 | No |
| 142 | 445501 | cdc26 |  |  | 17620 | -0.059 | 0.0176 | No |
| 143 | 117604 | ywhag1 |  |  | 18074 | -0.080 | 0.0039 | No |
| 144 | 560560\_1 | ywhag2 |  |  | 18090 | -0.080 | 0.0040 | No |
| 145 | 192333 | mcm7 |  |  | 18162 | -0.084 | 0.0024 | No |
| 146 | 567770 | atr |  |  | 18166 | -0.084 | 0.0029 | No |
| 147 | 558402 | anapc7 |  |  | 18470 | -0.097 | -0.0059 | No |
| 148 | 559665 | smc1a |  |  | 18605 | -0.104 | -0.0094 | No |
| 149 | 449548 | ppp2r1bb |  |  | 18698 | -0.108 | -0.0115 | No |
| 150 | 393885 | zgc:56064 |  |  | 18701 | -0.108 | -0.0108 | No |
| 151 | 30678 | pcna |  |  | 18983 | -0.121 | -0.0187 | No |
| 152 | 192302 | hdac1 |  |  | 19034 | -0.123 | -0.0194 | No |
| 153 | 559273\_2 | ep300a |  |  | 19072 | -0.125 | -0.0196 | No |
| 154 | 327060\_2 | wee1 |  |  | 19234 | -0.132 | -0.0237 | No |
| 155 | 336610 | ywhaz |  |  | 19338 | -0.137 | -0.0259 | No |
| 156 | 445386 | ndc80 |  |  | 19437 | -0.141 | -0.0280 | No |
| 157 | 406479 | orc5 |  |  | 19529 | -0.145 | -0.0298 | No |
| 158 | 338204 | tfdp2 |  |  | 19691 | -0.152 | -0.0337 | No |
| 159 | 393749 | tfdp1b |  |  | 19767 | -0.156 | -0.0349 | No |
| 160 | 30639 | smad2 |  |  | 19779 | -0.156 | -0.0341 | No |
| 161 | 100537961 | si:ch211-269e2.1 |  |  | 19825 | -0.158 | -0.0344 | No |
| 162 | 369195 | tgfb3 |  |  | 19890 | -0.161 | -0.0352 | No |
| 163 | 406419 | ywhabb |  |  | 19987 | -0.166 | -0.0370 | No |
| 164 | 399483 | cdkn1ca |  |  | 20283 | -0.178 | -0.0450 | No |
| 165 | 406816 | cul1b |  |  | 20667 | -0.197 | -0.0556 | No |
| 166 | 567111\_1 | crebbpb |  |  | 20683 | -0.198 | -0.0546 | No |
| 167 | 100318335 | anapc2 |  |  | 20894 | -0.209 | -0.0596 | No |
| 168 | 406610 | ppp2r5d |  |  | 21015 | -0.216 | -0.0618 | No |
| 169 | 324256 | orc6 |  |  | 21043 | -0.218 | -0.0610 | No |
| 170 | 359834 | tgfb1a |  |  | 21423 | -0.237 | -0.0712 | No |
| 171 | 569473 | anapc13 |  |  | 21458 | -0.238 | -0.0705 | No |
| 172 | 30222 | ccnd1 |  |  | 21522 | -0.242 | -0.0707 | No |
| 173 | 335195 | ywhaqb |  |  | 21570 | -0.244 | -0.0704 | No |
| 174 | 751740 | anapc15 |  |  | 21773 | -0.256 | -0.0748 | No |
| 175 | 406443 | ywhah |  |  | 21788 | -0.256 | -0.0734 | No |
| 176 | 794129 | mau2 |  |  | 22159 | -0.276 | -0.0830 | No |
| 177 | 403064 | atm |  |  | 22311 | -0.285 | -0.0856 | No |
| 178 | 58092 | smad3a |  |  | 22399 | -0.290 | -0.0862 | No |
| 179 | 394124 | cdc14aa |  |  | 22880 | -0.318 | -0.0990 | No |
| 180 | 559723\_1 | tgfb2l |  |  | 22957 | -0.324 | -0.0990 | No |
| 181 | 406740 | hdac8 |  |  | 23344 | -0.351 | -0.1085 | No |
| 182 | 557882 | si:dkeyp-80c12.7 |  |  | 23526 | -0.363 | -0.1115 | No |
| 183 | 373878 | ppp2r5eb |  |  | 24052 | -0.396 | -0.1252 | No |
| 184 | 794669\_1 | gb:cr929477 |  |  | 24180 | -0.405 | -0.1262 | No |
| 185 | 30188 | ccne1 |  |  | 24664 | -0.426 | -0.1382 | No |
| 186 | 449846 | rbx1 |  |  | 24797 | -0.438 | -0.1391 | No |
| 187 | 404206 | ccna1 |  |  | 24878 | -0.441 | -0.1383 | No |
| 188 | 569515 | cdk21 |  |  | 24908 | -0.441 | -0.1360 | No |
| 189 | 564982\_1 | mcm6l |  |  | 25025 | -0.448 | -0.1363 | No |
| 190 | 100331892 | rbl2 |  |  | 25425 | -0.478 | -0.1453 | No |
| 191 | 794108 | nipblb |  |  | 25471 | -0.482 | -0.1431 | No |
| 192 | 571082 | rad21l1 |  |  | 25692 | -0.502 | -0.1463 | No |
| 193 | 322060\_2 | ywhae1 |  |  | 25799 | -0.511 | -0.1459 | No |
| 194 | 327226\_1 | casc5 |  |  | 25887 | -0.519 | -0.1447 | No |
| 195 | 562014 | cdc16 |  |  | 25973 | -0.527 | -0.1435 | No |
| 196 | 568846 | si:ch211-160f23.5 |  |  | 26105 | -0.538 | -0.1436 | No |
| 197 | 100307076 | bub1 |  |  | 26647 | -0.592 | -0.1563 | No |
| 198 | 541325 | ccnh |  |  | 26657 | -0.593 | -0.1521 | No |
| 199 | 402825\_1 | cdc6 |  |  | 26773 | -0.606 | -0.1512 | No |
| 200 | 30637 | mdm2 |  |  | 26774 | -0.606 | -0.1467 | No |
| 201 | 560751 | stag2b |  |  | 26935 | -0.623 | -0.1471 | No |
| 202 | 503763 | ywhae2 |  |  | 27012 | -0.630 | -0.1448 | No |
| 203 | 336998 | chek2 |  |  | 27308 | -0.650 | -0.1492 | No |
| 204 | 449996 | ccnd3 |  |  | 27759 | -0.710 | -0.1581 | No |
| 205 | 405897 | cdk7 |  |  | 27781 | -0.713 | -0.1535 | No |
| 206 | 323299 | atrx |  |  | 27943 | -0.735 | -0.1531 | No |
| 207 | 777730 | cdk4 |  |  | 28245 | -0.777 | -0.1567 | No |
| 208 | 767751 | ccnb3 |  |  | 28375 | -0.800 | -0.1548 | No |
| 209 | 393223\_1 | fzr1a |  |  | 28391 | -0.802 | -0.1493 | No |
| 210 | 100333252 | cdkn2d |  |  | 29250 | -0.990 | -0.1690 | No |
| 211 | 100141368 | waplb |  |  | 29401 | -1.031 | -0.1661 | No |
| 212 | 322854 | anapc5 |  |  | 29637 | -1.107 | -0.1652 | No |
| 213 | 799015 | dbf4 |  |  | 29656 | -1.114 | -0.1574 | No |
| 214 | 406741 | e2f4 |  |  | 29707 | -1.132 | -0.1506 | No |
| 215 | 406462 | orc4 |  |  | 29738 | -1.144 | -0.1430 | No |
| 216 | 563708 | skp2 |  |  | 30286 | -1.382 | -0.1499 | No |
| 217 | 406813 | tfdp1a |  |  | 30317 | -1.397 | -0.1404 | No |
| 218 | 100307086 | pttg1 |  |  | 30318 | -1.398 | -0.1300 | No |
| 219 | 30590 | tp53 |  |  | 30325 | -1.401 | -0.1197 | No |
| 220 | 554509 | orc1 |  |  | 30327 | -1.402 | -0.1092 | No |
| 221 | 797406\_1 | espl1 |  |  | 30358 | -1.422 | -0.0995 | No |
| 222 | 324475\_1 | smc3 |  |  | 30490 | -1.500 | -0.0925 | No |
| 223 | 566841\_1 | crebbpa |  |  | 30571 | -1.563 | -0.0833 | No |
| 224 | 323883 | cul1a |  |  | 31083 | -2.063 | -0.0840 | No |
| 225 | 562282\_1 | ppp2r5b |  |  | 31087 | -2.070 | -0.0687 | No |
| 226 | 797406\_2 | espl1 |  |  | 31186 | -2.208 | -0.0553 | No |
| 227 | 431763\_1 | gadd45ab |  |  | 31309 | -2.449 | -0.0408 | No |
| 228 | novel.902 | - |  |  | 31511 | -2.951 | -0.0251 | No |
| 229 | 323299\_2 | atrx |  |  | 31877 | -4.954 | 0.0004 | No |
Table: GSEA details [plain text format]

  

Fig 2: CELL\_CYCLE(DRE04110)      
 Blue-Pink O' Gram in the Space of the Analyzed GeneSet

  

Fig 3: CELL\_CYCLE(DRE04110): Random ES distribution      
 Gene set null distribution of ES for **CELL\_CYCLE(DRE04110)**
